# Onychiurid species from Wanda Mountains in China, with descriptions of two new species (Collembola, Onychiuridae)

**DOI:** 10.3897/zookeys.425.7724

**Published:** 2014-07-15

**Authors:** Xin Sun, Donghui Wu

**Affiliations:** 1 Key laboratory of Wetland Ecology and Environment, Northeast Institute of Geography and Agroecology, Chinese Academy of Sciences, Changchun 130102, China; 2 Engineering Research Center of Chinese Ministry of Education for Edible and Medicinal Fungi, Jilin Agricultural University, Changchun 130118, China

**Keywords:** Taxonomy, new species, *Bionychiurus qinglongensis* sp. n., *Onychiurus heilongjiangensis* sp. n.

## Abstract

A checklist of onychiurid species from the Wanda Mountains in China is presented. Eighteen species belonging to twelve genera have been found, including two new species. *Bionychiurus qinglongensis* **sp. n.** can be easily distinguished from other known species of the genus by the absence of pseudocelli on Th. I tergum and fewer number of vesicles in postantennal organ. *Onychiurus heilongjiangensis* **sp. n.** is diagnosed by pseudocellar formulae as 32/133/33352 dorsally and 3/011/31120 ventrally, parapseudocellar formula as 0/000/111001+1^m^, ratio of anal spine/unguis as 0.6, unguiculus without inner basal lamella, and male ventral organ absent.

## Introduction

Heilongjiang Province is located in the northeast China, at the highest latitudes and the northernmost end of the country. It neighbors Russia across the Heilongjiang and Wusuli rivers running in its north and east respectively; in the west, it adjoins the Inner Mongolian Autonomous Region; and to its south is Jilin Province. It covers an area of 454,000 km^2^, accounting for 4.7% of the nation’s total. However, the fauna of Onychiuridae has been ignored in this area for many years. From 2011, we have investigated this group in Heilongjiang Province and reported several species gradually, including eleven species new to science and five new record species (Sun and Wu 2011; Sun et al. 2013a, b, c, d; Sun 2014). The Wanda Mountains are located in the eastern Heilongjiang Province of China, as a northern extension of the Changbai Mountain Range. During the investigations of the Collembola collected in this area, eighteen onychiurid species belonging to twelve genera have been found, including two new species, *Bionychiurus qinglongensis* sp. n. and *Onychiurus heilongjiangensis* sp. n. In the present work, a checklist of the onychiurid species from Wanda Mountains is presented and descriptions of the two new species are given.

## Material and methods

Eight sampling sites of Wanda Mountains have been studied, which are all planted forest with *Quercus mongolica* Fisch. ex Ledeb., *Pinus koraiensis* Sieb. et Zucc., *Betula costata* Trautv. or *Populus davidiana* Dode (Table 1, Fig. 5). Specimens were collected by Berlese extraction, cleared in lactic acid and then mounted in Marc André II solution. They were studied using a Nikon Eclipse 80i microscope. The material is deposited in the Key Laboratory of Wetland Ecology and Environment, Northeast Institute of Geography and Agroecology, Chinese Academy of Sciences, Changchun.

**Table 1.** List of the sampling sites of Wanda Mountains in China.

No.	Name of the site	City	County	Coordinates	Altitude	Habitats	Date	Collector
1	Jiejinshan	Jiamusi	Tongjiang	47.9185°N, 132.8503°E	95 m	Litter and soil under *Quercus mongolica Fisch. ex Ledeb.*	7 Aug. 2010	Donghui Wu et al.
2	Qiyuan	Shuangyashan	Raohe	46.6295°N, 133.4319°E	155 m	Litter and soil under *Pinus koraiensis Sieb. et Zucc.*	16 Aug. 2010	Donghui Wu et al.
3	Wulindong	Shuangyashan	Raohe	46.5650°N, 133.6690°E	207 m	Litter and soil under *Pinus koraiensis Sieb. et Zucc.*	18 Sept. 2011	Haitao Wu and Lihong Song
4	Zhenbaodao	Shuangyashan	Raohe	46.4882°N, 133.8454°E	75 m	Litter and soil under *Pinus koraiensis Sieb. et Zucc.*	16 Aug. 2010	Donghui Wu et al.
5	Shendingshan	Shuangyashan	Raohe	46.4760°N, 133.3031°E	159 m	Litter and soil under *Pinus koraiensis Sieb. et Zucc.*	16 Aug. 2010	Donghui Wu et al.
6	Qinglongshan	Shuangyashan	Baoqing	46.1504°N, 131.9591°E	259 m	Litter and soil under *Betula costata Trautv.*	14 Aug. 2010	Donghui Wu et al.
7	Bawu’er	Shuangyashan	Baoqing	46.1370°N, 132.8580°E	149 m	Litter and soil under *Populus davidiana Dode*	15 Aug. 2010	Donghui Wu et al.
8	Hulin	Jixi	Hulin	45.7633°N, 133.0453°E	91 m	Litter and soil under *Quercus mongolica Fisch. ex Ledeb.*	15 Aug. 2010	Donghui Wu et al.

Labial types are determined after Fjellberg (1999). Areas and chaetal nomenclature of labium follow Massoud (1967) and D’Haese (2003). Chaetae on anal valves are identified after Yoshii (1996). Chaetae on the furcal area are classified in accordance with Weiner (1996). Tibiotarsus chaetotaxy formula follows Deharveng (1983), and is expressed as: total number of chaetae (number of chaetae in row C, number of chaetae in row B, number of chaetae in distal rows A+T), for example 22 (3, 8, 11).

### Abbreviations used in descriptions

Ant. – antennal segments, PAO – postantennal organ, Th. – thoracic segments, Abd. – abdominal segments, ms – microsensillum, pso – pseudocellus, psx – parapseudocellus, Sp – posterior S-chaeta on Abd. V tergum, m – unpaired pseudopore or parapseudocellus.

The pseudocelli, parapseudocelli and pseudopores formula are the number of pseudocelli, parapseudocelli, or pseudopores by half-tergum (dorsally) or half-sternum (ventrally) as follows: head anterior, head posterior/Th. I, II, III/Abd. I, II, III, IV, V (for instance: 32/033/33343).

## Systematics

### Checklist of onychiurid species from Wanda Mountains in China (see Table 1 for details on sample sites)

*Allonychiurus songi* Sun & Wu, 2012

Locality. Bawu’er, Hulin, Jiejinshan, Qiyuan, Qinglongshan, Shendingshan, Zhenbaodao.

*Bionychiurus qinglongensis* sp. n.

Locality. Qinglongshan.

*Bionychiurus changbaiensis* Sun & Wu, 2012

Locality. Zhenbaodao.

*Dimorphaphorura sanjiangensis* Sun & Wu, 2012

Locality. Bawu’er, Qinglongshan, Shendingshan.

*Heteraphorura seolagensis* (Lee, 1974)

Locality. Hulin, Jiejinshan, Qiyuan, Qinglongshan, Zhenbaodao.

*Hymenaphorura maoerensis* Sun, 2014

Locality. Jiejinshan.

*Hymenaphorura wusuliensis* Sun & Wu, 2011

Locality. Wulindong.

*Oligaphorura chankaensis* Sun & Wu, 2012

Locality. Shendingshan.

*Oligaphorura koreana* (Weiner, 1994)

Locality. Jiejinshan, Qiyuan, Zhenbaodao.

*Oligaphorura ursi* Fjellberg, 1984

Locality. Hulin, Shendingshan.

*Onychiurus heilongjiangensis* sp. n.

Locality. Qinglongshan, Jiejinshan.

*Protaphorura bicampata* (Gsin, 1956)

Locality. Hulin.

*Protaphorura minima* Sun, Zhang & Wu, 2013

Locality. Jiejinshan, Qiyuan, Qinglongshan.

*Protaphorura nutak* (Yosii, 1972)

Locality. Zhenbaodao.

*Psyllaphorura raoheensis* Sun & Wu, 2011

Locality. Bawu’er, Qinglongshan, Shendingshan, Wulindong, Zhenbaodao.

*Sensillonychiurus changchunensis* Sun & Wu, 2012

Locality. Shendingshan.

*Supraphorura furcifera* (Börner, 1901) new record

Locality. Zhenbaodao.

*Thalassaphorura macrospinata* Sun & Wu, 2012

Locality. Hulin, Jiejinshan, Qinglongshan, Zhenbaodao.

*Thalassaphorura problematica* Sun, Deharveng & Wu, 2013

Locality. Wulindong, Zhenbaodao.

### Bionychiurus qinglongensis sp. n.

Taxon classificationAnimaliaORDOFAMILIA

http://zoobank.org/2811C6C2-4DCD-43EA-8A52-CF3210DB03F0

Figures 1 –2

#### Type material.

Holotype male, two female paratypes on slides – China, Heilongjiang, Shuangyashan, Baoqing, Qinglongshan (46.1504°N, 131.9591°E), 14.Aug.2010, litter and soil under *Betula costata* Trautv., Berlese extraction, Wu Donghui et al. leg. (LD-10-344).

#### Diagnosis.

Pso formulae as 32/033/33343 dorsally and 11/000/01(0)010 ventrally. Subcoxae 1 of legs I, II and III with 1, 1 and 1 pso respectively. Psx formula as 00/000/100001^m^ ventrally. PAO with 14–17 compound vesicles. Th. I–III sterna with 0+0, 1+1, 1+1 chaetae respectively. Ventral tube with 1+1 basal chaetae. Subcoxae 1 of legs I, II and III with 5, 7 and 6 chaetae respectively.

#### Description.

Body length: females 1.30–1.37 mm, male holotype 1.08 mm. Shape of body typical of the genus: cylindrical with strong anal spines on papillae. Color in alcohol white.

**Figure 1.**
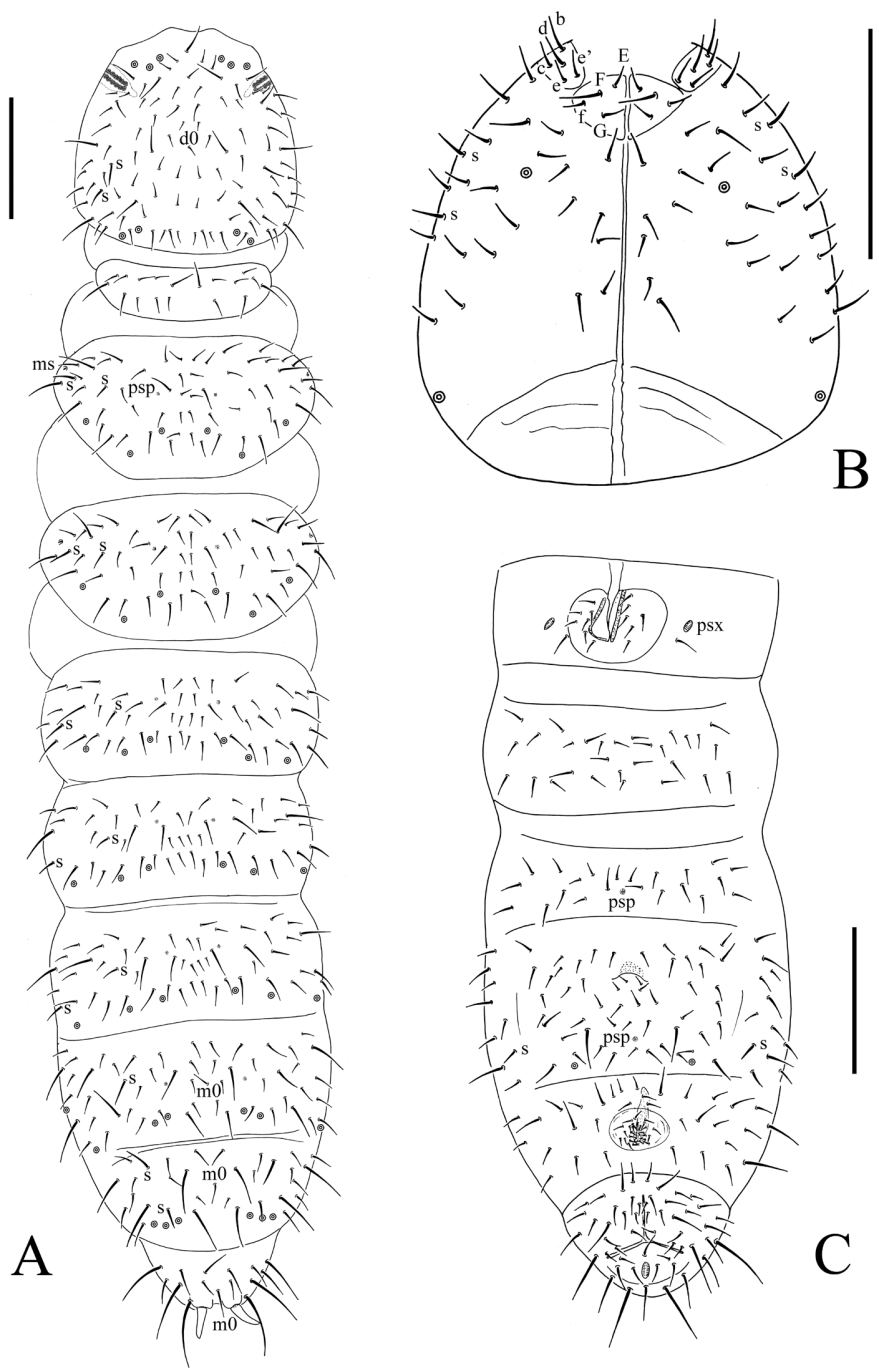
*Bionychiurus qinglongensis* sp. n. **A** dorsal side of body **B** ventral side of head **C** general view of Abd. I–VI sterna. Scale bars: 0.1 mm (**A–C**).

Pso formulae: 32/033/33343 dorsally and 11/000/01(0)010 ventrally (Figs 1A, B, C). Subcoxae 1 of legs I–III with 1 pso each. Parapseudocellar formulae: 00/000/100001^m^ ventrally, dorsally psx absent (Figs 1A, B, C). Pseudopore formulae: 00/011/11110 dorsally and 00/111/001^m^1^m^ 0 ventrally (Figs 1A, B, C).

**Figure 2.**
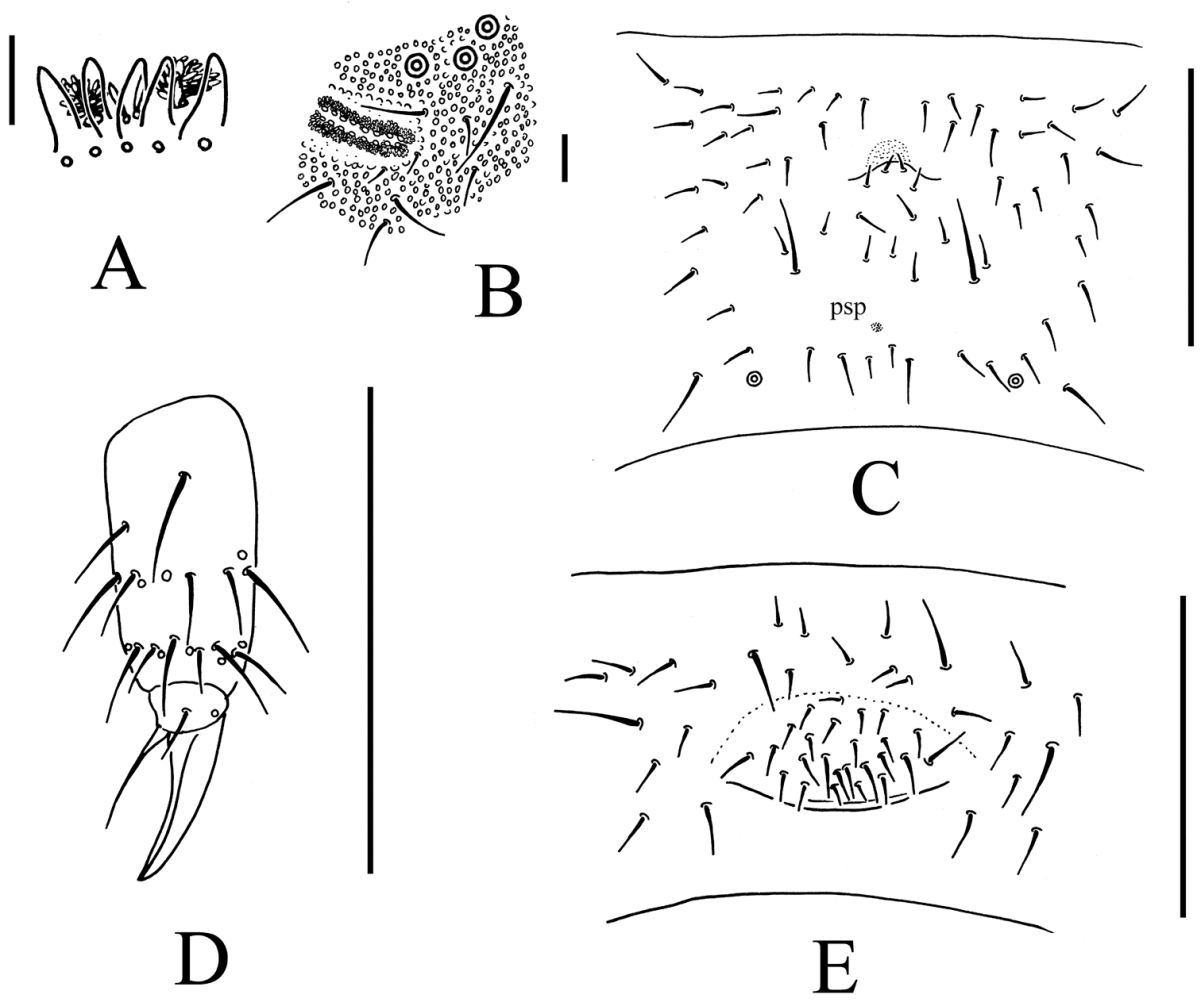
*Bionychiurus qinglongensis* sp. n. **A** Ant. III sensory organ **B** PAO **C** Abd. IV sternum **D** distal part of leg III **E** female genital plate. Scale bars: 0.1 mm (**C–E**), 0.01 mm (**A–B**).

Head. Antennae short, 1.2 times as long as head. Length ratio of antennal segments I : II : III : IV = 1 : 1.5 : 1.5 : 2. Subapical organite on Ant. IV with globular apex; basolateral ms above the second proximal row of chaetae. Ant. III sensory organ consisting of 5 papillae, 5 guard chaetae, 2 small sensory rods, 2 granulated sensory clubs and a later ms (Fig. 2A). Ant. II with 15 chaetae. Ant. I with 10 chaetae. Antennal base weakly marked. PAO with 14–17 compound vesicles arranged in two rows along axis of organ (Fig. 2B). Dorsal cephalic chaeta d0 present. 4+4 p-chaetae between posterior a-pso on head, p1 in line with others (Fig. 1A). Mandible with strong molar plate and 4 apical teeth. Maxilla bearing 3 teeth and 6 lamellae. Maxillary palp simple with 1 basal chaeta and 2 sublobal hairs. Labral chaetal formula 4/342. Labium with 6 proximal, 4 basomedian (E, F, G and f), and 5 basolateral chaetae (b, c, d, e, e’) (Fig. 1B); labial palp of AC type, labial papillae A–E with 1, 4, 0, 3, and 3 guard chaetae respectively. 5+5 postlabial chaetae along ventral groove (Fig. 1B).

Body chaetotaxy. S-chaetae formulae as 11/022/222120 dorsally and 11/000/000100 ventrally (Figs 1A, B, C). Tiny and blunt ms, present on Th. II–III dorsal-laterally (Fig. 1A). Dorsal ordinary chaetae differentiated, Sp : m1 : p1 ratio on Abd. V = 1 : 0.8 : 2.5 (Fig. 1A). Th. I tergum with 9–12 chaetae. Th. II–III terga with 5+5 and Abd. I–III terga with 3–4+3–4 chaetae on both side of axial line respectively and no unpaired axial chaetae (Fig. 1A). On each other abdominal terga from Abd. IV to Abd. VI tergum axial chaetae m0 present (Fig. 1A). Th. I–III sterna with 0+0, 1+1, 1+1 chaetae between legs respectively.

Appendages. Subcoxae 1 of legs I, II and III with 5, 7 and 6 chaetae, subcoxae 2 with 1, 5 and 5 chaetae respectively. Coxae of legs I, II and III with 4, 11 and 14 chaetae, trochanters with 10 chaetae each and femora with 15, 17 and 17 chaetae. Tibiotarsi of legs I, II and III with 22 (3, 8, 11), 20 (1, 8, 11) and 21 (2, 8, 11) chaetae (Fig. 2C). Unguis without teeth. Unguiculus slender and pointed, 0.8–0.9 times as long as inner edge of unguis, without inner basal lamella (Fig. 2C). Ventral tube with 6–9+6–9 distal chaetae and 1+1 basal chaetae, without anterior chaetae (Fig. 1C). Furca reduced to cuticular fold with 4 small dental chaetae posteriorly and two manubrial rows of chaetae (Fig. 2B)

Genital plate with 16–25 chaetae in females (Fig. 2D), 26 in male. Male ventral organ absent. Anal valves with numerous acuminate chaetae; each lateral valve with a0, 2a1 and 2a2; upper valve with chaetae a0, 2a1, 2b1, 2b2, c0, 2c1 and 2c2 (Fig. 1C). Anal spines set on distinct papillae, as long as inner edge of hind unguis (Fig. 1A).

#### Derivatio nominis.

Named for the name of mountain (Qinglongshan, and -shan means mountain in Chinese) where the species was found.

#### Discussion.

Until now, there are four known species belonging to the genus *Bionychiurus*: *Bionychiurus changbaiensis* Sun & Wu, 2012 from China, *Bionychiurus normalis* (Gisin, 1949) from Europe, *Bionychiurus oblongatus* (Lee & Park, 1986), and *Bionychiurus yongyeonensis* (Yosii, 1966) from South Korea (Bellinger et al. 1996–2014; Sun and Wu 2012). The new species can be easily distinguished from all the above mentioned congeners by the absence of pso on Th. I tergum (present in other species) and fewer number of vesicles in PAO (more than 17 in other species).

### Onychiurus heilongjiangensis sp. n.

Taxon classificationAnimaliaORDOFAMILIA

http://zoobank.org/540034F0-0A0F-4599-9E38-91BDC042C420

Figures 3 –4

#### Type material.

Holotype male, 7 female and 6 male paratypes on slides – China, Heilongjiang, Shuangyashan, Baoqing, Qinglongshan (46.1504°N, 131.9591°E), 14.Aug.2010, litter and soil under *Betula costata* Trautv., Berlese extraction, Wu Donghui et al. leg. (LD-10-444, LD-10-445); 12 female and 3 male paratypes on slides – China, Heilongjiang, Jiamusi, Tongjiang, Jiejinshan (47.9185°N, 132.8503°E), 7.Aug.2010, litter and soil under *Quercus mongolica* Fisch. ex Ledeb., Berlese extraction, Wu Donghui et al. leg. (LD-10-484, LD-10-485, LD-10-486, LD-10-488).

**Diagnosis.** Pso formulae as 32/133/33352 dorsally and 3/011/31120 ventrally. Psx formula as 0/000/111001+1^m^ ventrally.. Ratio of AS/unguis as 0.6. Unguiculus without inner basal lamella. Male ventral organ absent.

#### Description.

Body length: females 1.6–1.8 mm, males 1.4–1.6 mm; holotype 1.6 mm. Shape of body typical of the genus: cylindrical with anal spines on papillae, Abd. III–IV more or less broadened. Color in alcohol white.

**Figure 3.**
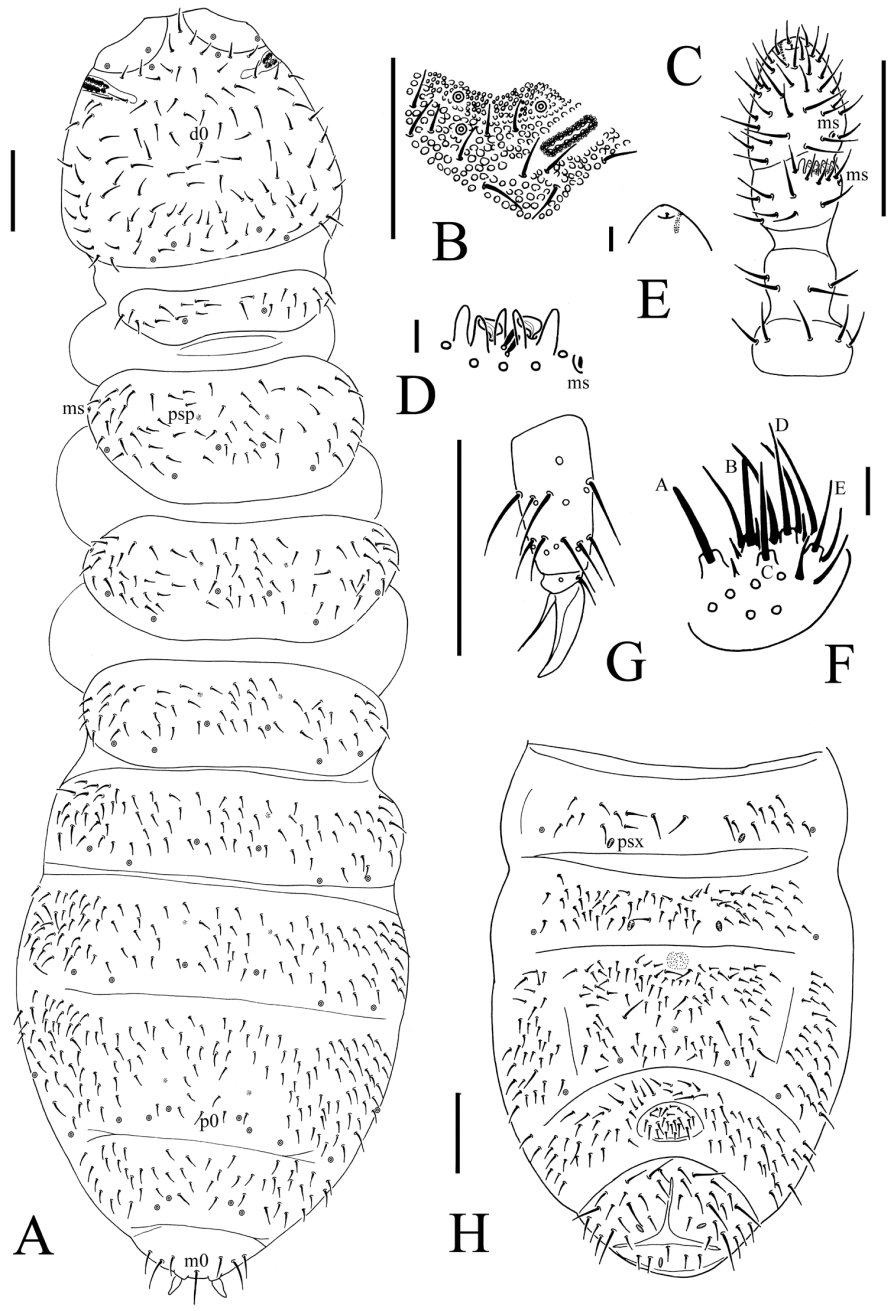
*Onychiurus heilongjiangensis* sp. n. **A** dorsal side of body **B** PAO **C** Ant. I–IV **D** Ant. III sensory organ **E** antennal tip **F** labium **G** distal part of leg III **H** Abd. II–VI sterna. Scale bars: 0.1 mm (**A, C, G–H**), 0.01 mm (**B, D–F**).

**Figure 4.**
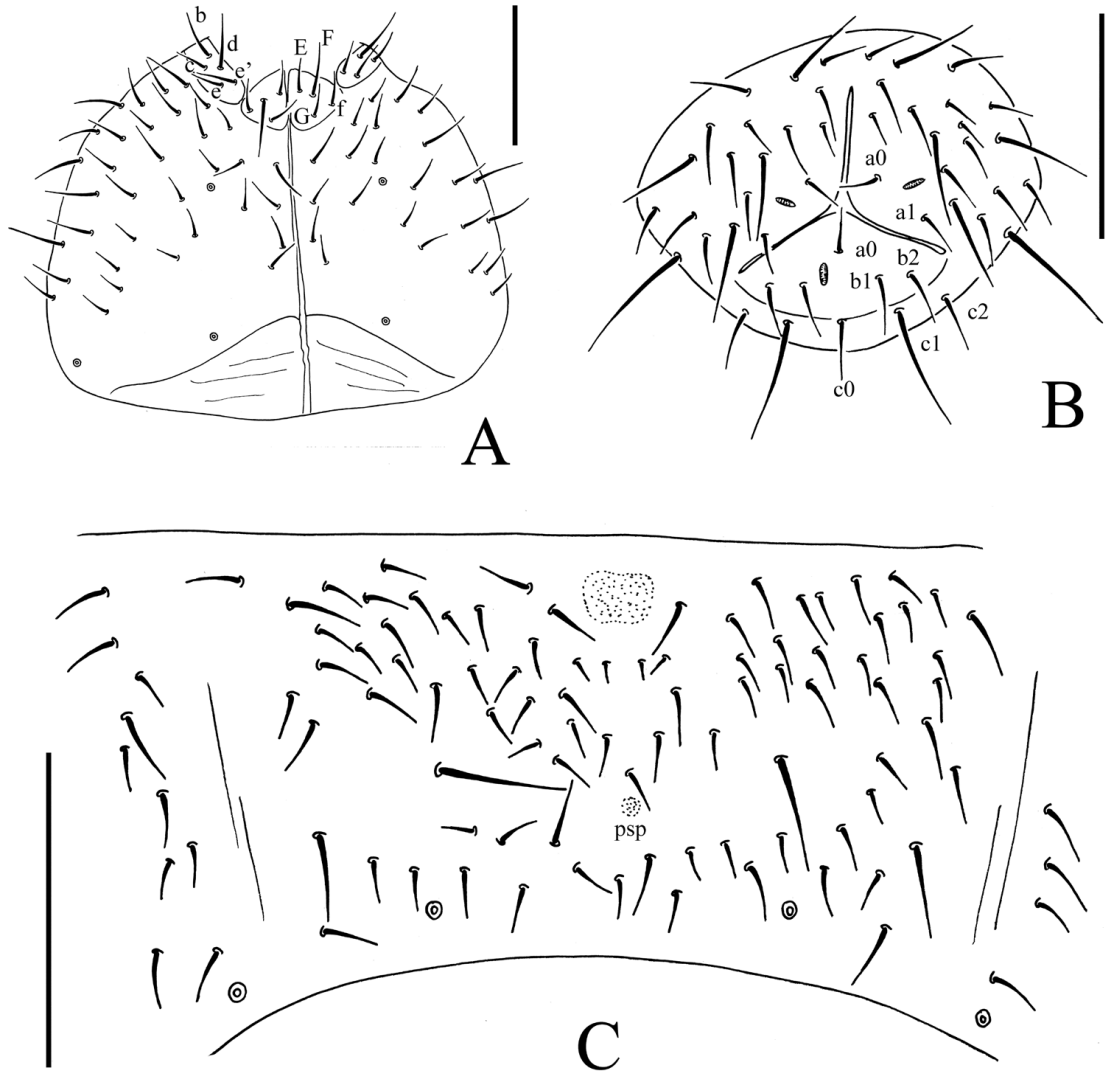
*Onychiurus heilongjiangensis* sp. n. **A** ventral side of head **B** anal valves **C** Abd. IV sternum. Scale bars: 0.1 mm (**A–C**).

Pso formulae: 32/133/33352 dorsally and 3/011/31120 ventrally (Figs 3A, H, 4A). Subcoxae 1 of legs I–III with 2 pso each. Parapseudocellar formulae: 0/000/111001+1^m^ ventrally, dorsally psx absent (Figs 3A, H, 4A). Pseudopore formulae as 00/011/11110 dorsally and 00/111/0001^m^00 ventrally (Figs 3A, H, 4A).

Head. Antennae short, as long as head. Length ratio of antennal segments I : II : III : IV = 1 : 1.5 : 1.5 : 2.0. Subapical organite on Ant. IV with globular apex (Fig. 3C); invaginated apical bulb present (Fig. 3E); basolateral ms above the first proximal row of chaetae (Fig. 3C). Ant. III sensory organ composed of 5 papillae, 5 guard chaetae, 2 small rods, and 2 smooth sensory clubs (Fig. 3D); lateral ms just behind sensory organ (Fig. 3C). Ant. II with 14 chaetae. Ant. I with 8 chaetae. Antennal base well marked. PAO with 14–16 compound vesicles arranged in two rows along axis of organ (Fig. 3B). Dorsal cephalic chaeta d0 present. 3+3 p-chaetae between two inner posterior pso on head, p1 anterior to others (Fig. 3A). Mandible with strong molar plate and 4 apical teeth. Maxilla bearing 3 teeth and 6 lamellae. Maxillary palp simple with 1 basal chaeta and 2 sublobal hairs. Labral chaetae 4/142. Labium with 6 proximal, 4 basomedian (E, F, G, and f) and 5 basolateral (b, c, d, e, e’) chaetae (Fig. 4A). Labial palp of AB type, labial papillae A–E with 1, 4, 0, 3 and 3 guard chaetae respectively (Fig. 3F). Postlabial chaetae 4–5+4–5 along ventral groove (Fig. 4A).

Body chaetotaxy. S-chaetae not distinguishable from ordinary chaetae. Tiny and blunt ms, present on Th. II and III dorso-laterally (Fig. 3A). Dorsal ordinary chaetae poorly differentiated, usually coarse and short. Th. I tergum with 8–14+8–14 chaetae. Th. II–Abd. III terga with 4–5+4–5 chaetae along axial line respectively, usually with asymmetrical chaetae (Fig. 3A). Abd. IV tergum with p0 chaeta; Abd. V tergum without axial chaeta; Abd. VI tergum with m0 chaeta (Fig. 3A). Th. I–III sterna without chaetae between legs.

Appendages. Subcoxae 1 of legs I–III with 5, 5 and 5 chaetae, subcoxae 2 with 1, 4 and 4 chaetae respectively. Coxae of legs I, II and III with 3, 9–11 and 9–12 chaetae, trochanters with 9 chaetae each and femora with 15, 16 and 16 chaetae. Tibiotarsi of legs I, II and III with 17 (9, 7, 1), 18 (9, 7, 2) and 17 (9, 7, 1) chaetae, M-chaeta absent. Unguis without teeth. Unguiculus slender and pointed, 0.8 times as long as inner edge of unguis, without inner basal lamella (Fig. 3G). Ventral tube with 6–8+6–8 distal chaetae, without anterior or basal chaetae. Furca reduced to finely granulated area, with 4 small dental chaetae in one row posteriorly; three manubrial rows of chaetae present (Figs 3H, 4C).

Genital plate with 17–23 chaetae in females, 40–63 chaetae in males. Male ventral organ absent. Anal valves with numerous acuminate chaetae; each lateral valve with chaetae a0 and 2a1; upper valve with chaetae a0, 2b1, 2b2, c0, 2c1 and 2c2 (Fig. 4B). Anal spines set on distinct papillae, 0.6 times as long as inner edge of hind unguis (Fig. 3A).

#### Derivatio nominis.

Named for the province of the type locality.

#### Discussion.

The new specie shares the same dorsal pso formula from head to Abd. IV (32/133/3335), ventral pso formula from head to Abd. I (3/011/3) and number of pso on subcoxae 1 of legs I–III (2, 2, 2 respectively) with a number of the known European species, i.e. *Onychiurus ambulans* (Linnaeus, 1758) sensu Pomorski, 1998, *Onychiurus arans* Gisin, 1952, *Onychiurus circulans* Gisin, 1952, *Onychiurus insinuans* Gisin, 1952, *Onychiurus subcirculans* Gisin, 1962, and *Onychiurus sublegans* Gisin, 1960, but it can be distinguished easily from all these species as having only 2 pso on each side of Abd. V (3 or 4 in other species) and no male ventral organ (present in other species).

**Figure 5.**
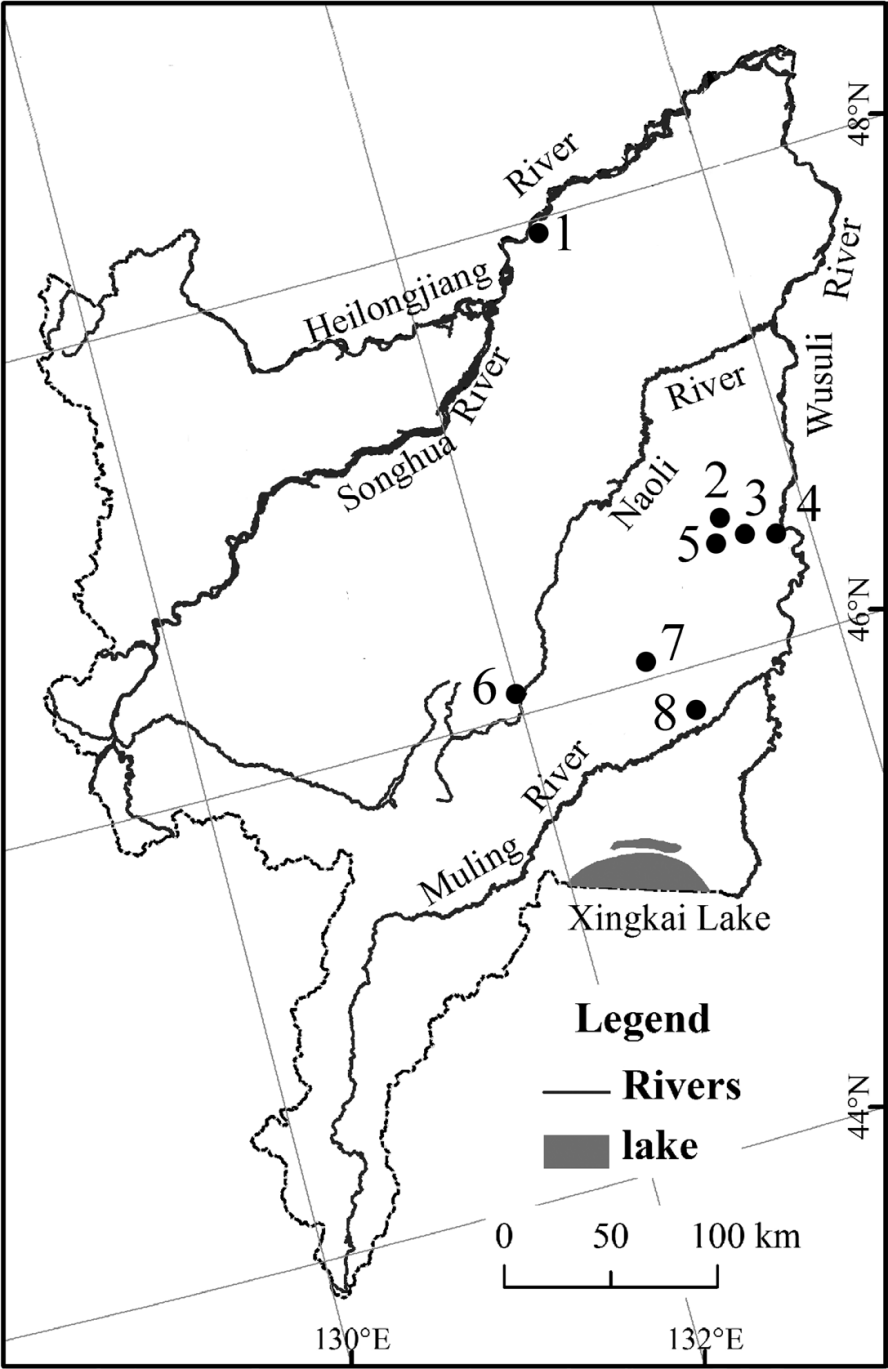
Sampling localities in Wanda Mountains.

## Supplementary Material

XML Treatment for Bionychiurus qinglongensis

XML Treatment for Onychiurus heilongjiangensis
